# Supplementary material for: ANGPTL8 negatively regulates NF-κB activation by facilitating selective autophagic degradation of IKKγ
Source: Nat Commun. 2017 Dec 18;8:2164. doi: 10.1038/s41467-017-02355-w (PMC5735157; doi:10.1038/s41467-017-02355-w)
Supplement: Supplementary file 1 — Supplementary Information [file 41467_2017_2355_MOESM1_ESM.pdf]

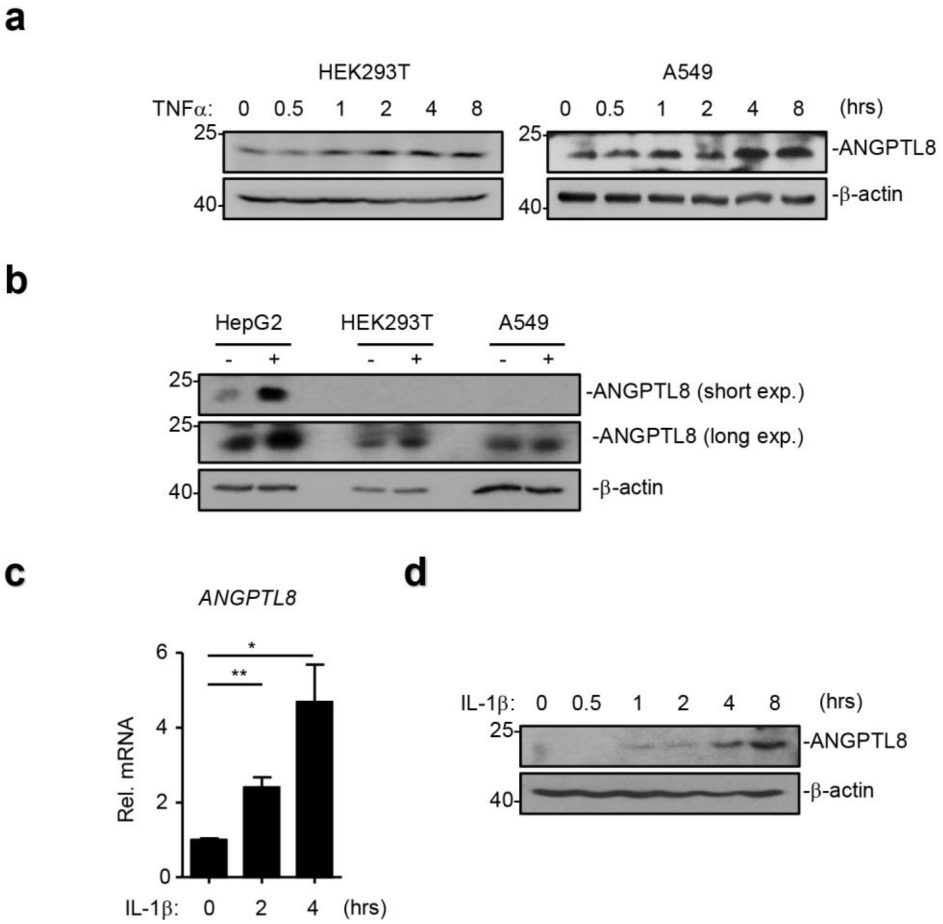

Supplementary Figure 1. TNFα and IL-1β up-regulate the expression of ANGPTL8. a. The expression level of ANGPTL8 after TNFα treatment. **b.** The induction of ANGPTL8 in TNFα-induced HEK293T or A549 cells. **c,d.** The transcription level (***c***, n=3) and protein level ***(d)*** of ANGPTL8 after IL-1β (10 ng/mL) treatment. Data are shown as mean ± SEM, unpaired two-tailed student's test was used for statistics ***(c)***, *p < 0.05, **p < 0.01. Data are representative of three independent experiments.

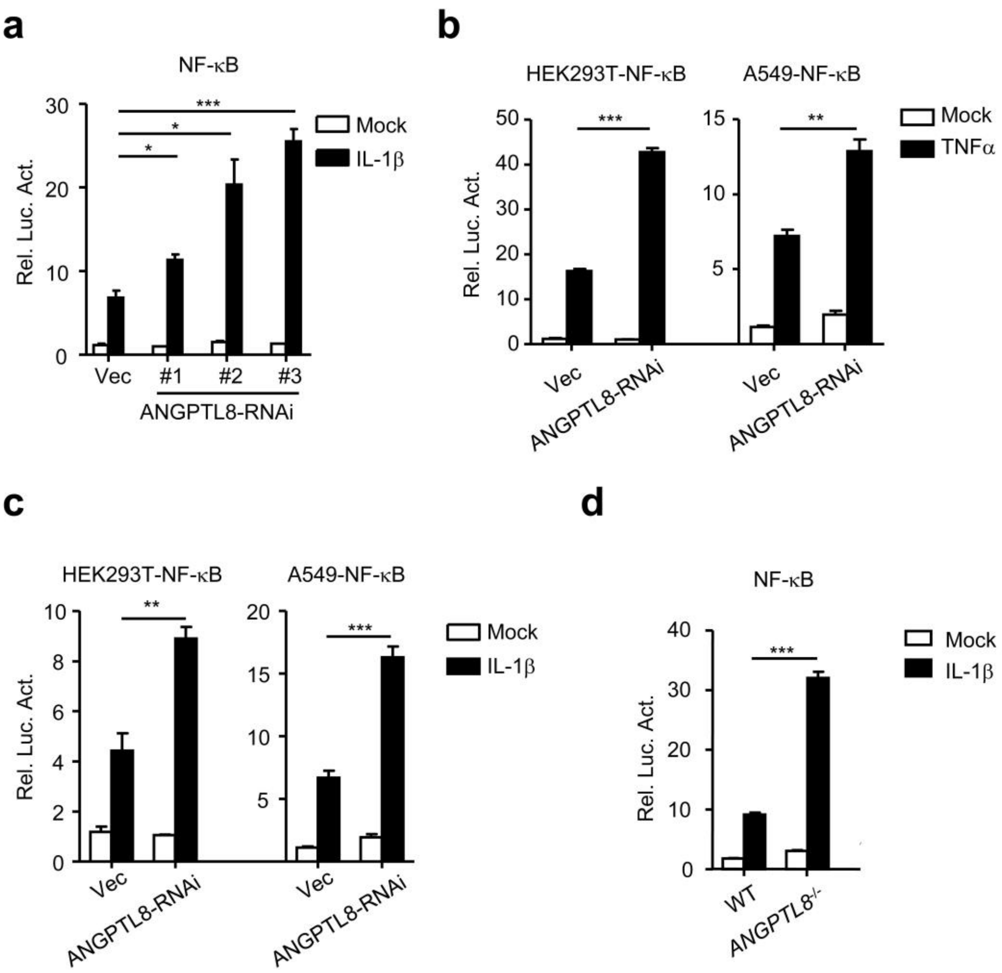

Supplementary Figure 2. Knockdown or knockout of ANGPTL8 potentiates TNFα- or IL-1β-induced NF-κB activation. a. Effects of ANGPTL8-RNAi on IL-1β-induced NF-κB activation in HepG2 cells (n=3). **b,c.** Effects of ANGPTL8-RNAi on TNFα- ***(b)*** or IL-1β- ***(c)*** induced NF-κB activation in HEK293T and A549 cells (n=3). **d.** Effects of ANGPTL8 deficiency on IL-1β-induced NF-κB activation. Data are shown as mean ± SEM, unpaired two-tailed student's test was used for statistics, *$p < 0.05$, **$p < 0.01$, ***$p < 0.001$. Data are representative of three independent experiments.

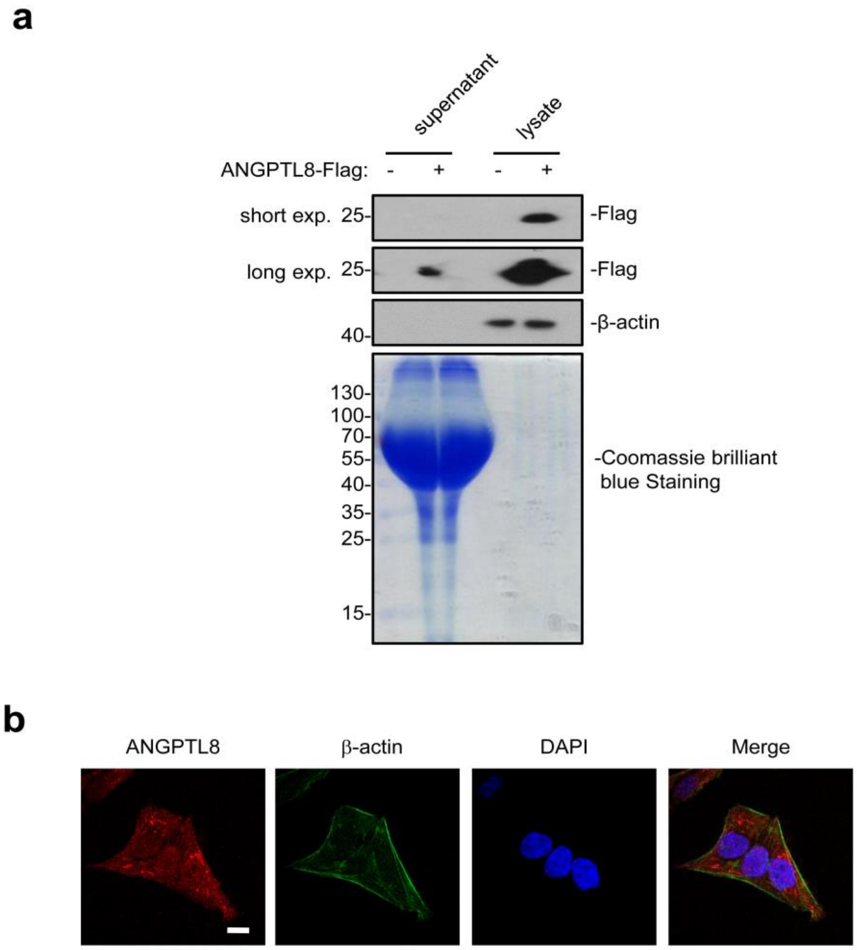

Supplementary Figure 3. The cellular localization of ANGPTL8. a. The expression of ANGPTL8 in the supernatant and lysate following gene transfection in HepG2 cells. The HepG2 cells were transfected with control vector or ANGPTL8-Flag plasmids for 48 hours, and then medium in one well of 6-well plates was precipitated with 20% trichloroacetic acid and resuspended by 150 μl RIPA buffer (supernatant), while cell pellet in the same well were lyzed by 150 μl RIPA buffer directly (lysate). For immunoblots, 5 μl of lysate or 25 μl of supernatant was applied, respectively. **b.** The immunofluorescence analysis of the cellular localization of endogenous ANGPTL8 protein in HepG2 cells. Data are representative of three independent experiments.

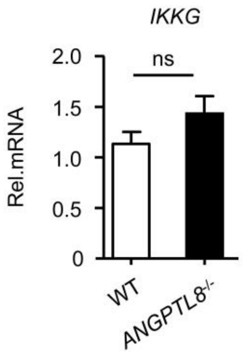

Supplementary Figure 4. Effects of ANGPTL8 deficiency on the transcription of IKKγ. *ANGPTL8*$^{-/-}$ and control cell lines were lysed and then total RNA was extracted for the detection of *IKKG* mRNA. Data are shown as mean ± SEM, unpaired two-tailed student's test was used for statistics, n=3, ns: p>0.05. Data are representative of three independent experiments.

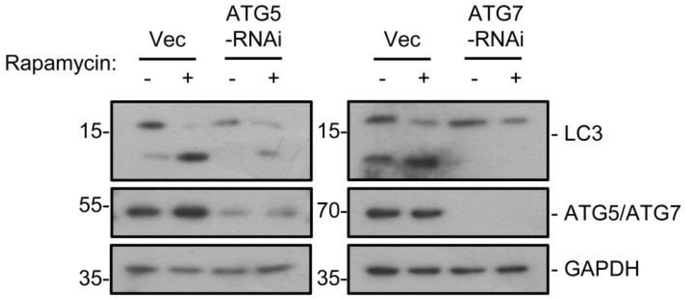

Supplementary Figure 5. Effects of rapamycin-induced LC3 turnover in the control or ATG5/7-RNAi cell lines. ATG5/7-RNAi or control HEK293T cell lines were treated with rapamycin (0.5 μg/ml) for 12 hours, then cells were harvested and subjected to immunoblots.

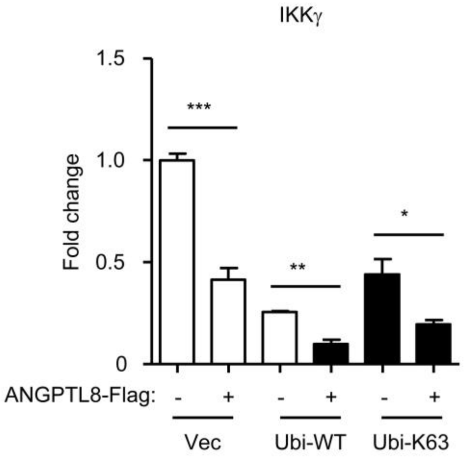

Supplementary Figure 6. Quantitative densitometric results of the effects of ubiquitin on the ANGPTL8-mediated IKKγ degradation. GAPDH was used as the loading control. Data are shown as mean± SEM, unpaired two-tailed student's test was used for statistics, n=3 independent experiments, *p < 0.05, **p < 0.01, ***p < 0.001.

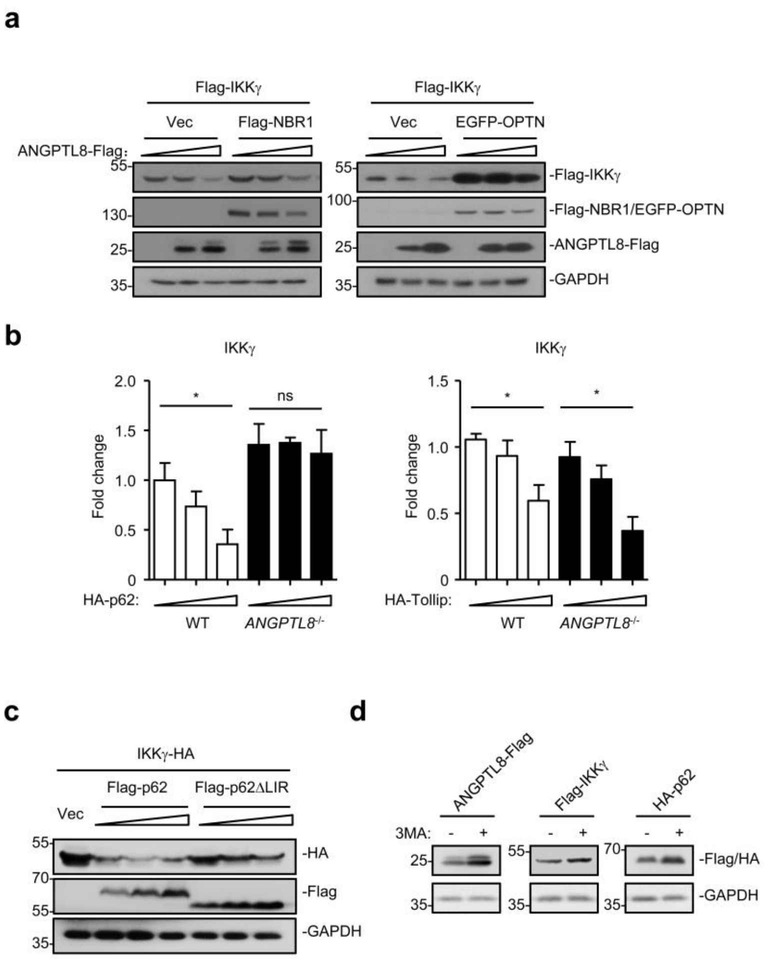

Supplementary Figure 7. Autophagy receptor p62 mediates IKKγ degradation in an ANGPTL8-dependent manner. a. Autophagy adaptor NBR1 and OPTN do not mediate the degradation of IKKγ in HEK293T cells. **b.** The quantitative densitometric results of the overexpression of p62- and Tollip- mediated IKKγ degradation in *ANGPTL8*$^{-/-}$ or control cells. GAPDH was used as the loading control (n=3 independent experiments). **c.** The effects of the overexpression of full length and ΔLIR truncation mutants of p62-mediated IKKγ degradation. **d.** Overexpressed ANGPTL8, IKKγ and p62 degrade via autophagy in HEK293T cells. Cells were transfected with indicated plasmids for 24 hours before treated by 3MA (335 μM) for 6 hours. Data are shown as mean ± SEM, unpaired two-tailed student's test was used for statistics ***(b)***, $*p < 0.05$, ns: $p>0.05$. Data are representative of three independent experiments.

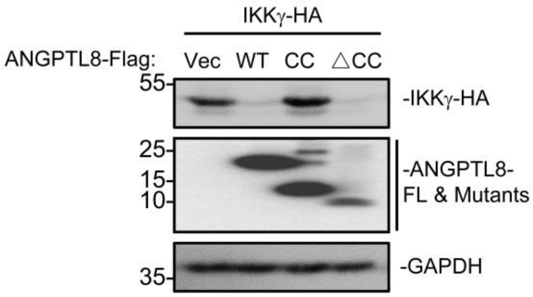

Supplementary Figure 8. Effects of ANGPTL8 coiled-coil (CC) domain on the ANGPTL8-mediated IKKγ degradation. HEK293T cells were transfected with indicated plasmids for 24 hours and immunoblots were performed with the indicated antibodies. Data are representative of three independent experiments.

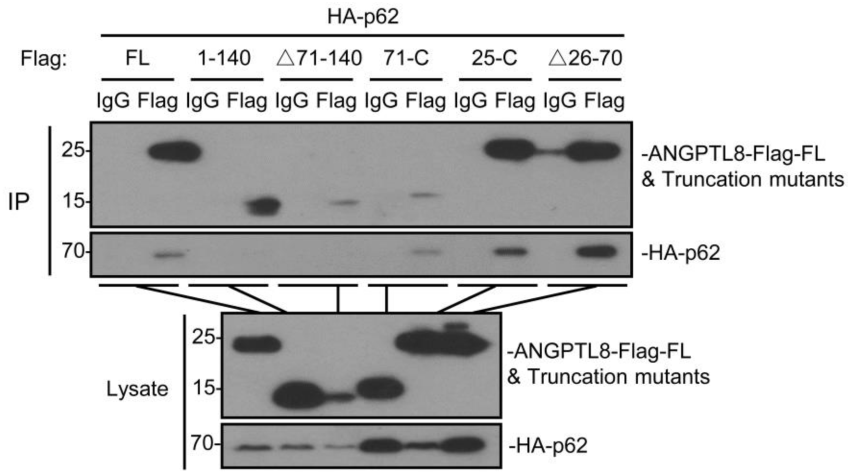

Supplementary Figure 9. Domain mapping of the interaction between ANGPTL8 and p62. HEK293T cells were transfected with indicated plasmids for 24 hours. Co-IP analysis and immunoblots were performed with the indicated antibodies. Data are representative of three independent experiments.

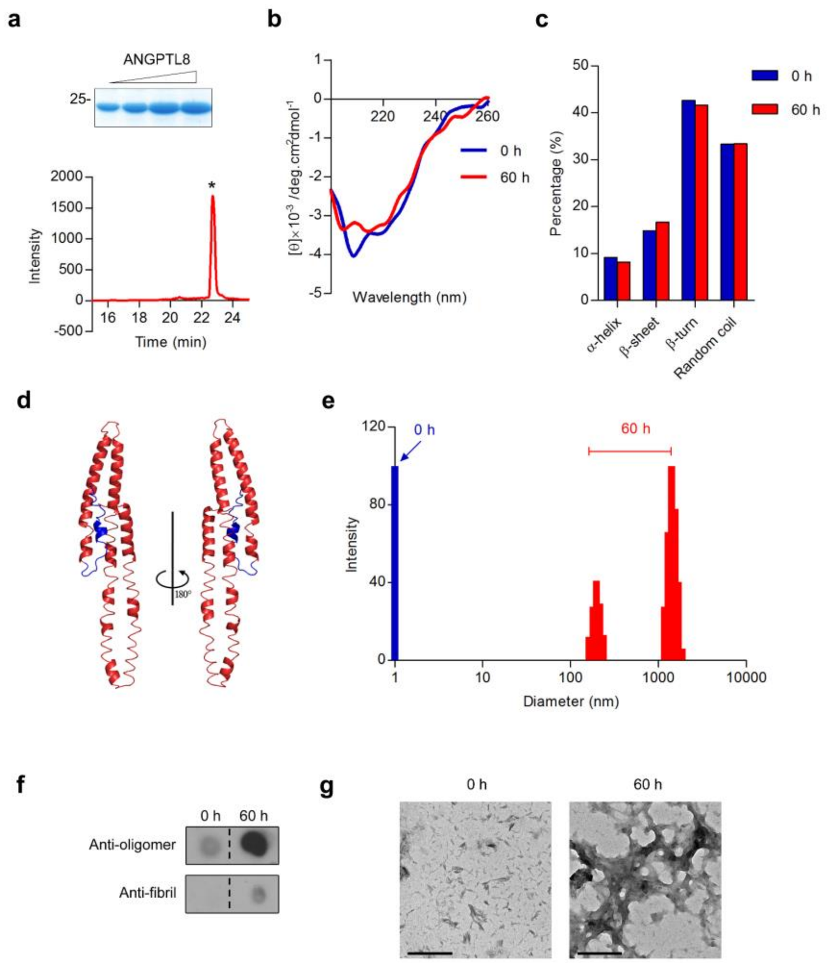

Supplementary Figure 10. The aggregation and structural characteristics of ANGPTL8. **a.** SDS-PAGE of expressed ANGPTL8 (*up* panel) and RP-HPLC purification of ANGPTL8 (*bottom* panel). **b.** Far-UV circular dichroism spectra of ANGPTL8 (incubated in 25 mM PBS, pH 7.4, containing 50 mM NaCl) at 37℃ for 0 and 60 hours, the percentage of secondary structure was quantified with CDPro and shown in (***c***). **d.** The simulated structure of ANGPTL8, blue represents residues 1-25, red represents residues 26-198; **e.** The average particle diameters of ANGPTL8 aggregates generated upon incubation (0 and 60 hours; blue and red, respectively) measured by dynamic light scattering. **f.** The oligomerization and fibrillation of ANGPTL8 detected by dot blot using A11 and OC antibody, respectively. **g.** TEM image of ANGPTL8 incubated for 0 and 60 hours, scale bar represents 200 nm. Data are representative of three independent experiments (**b,e**).

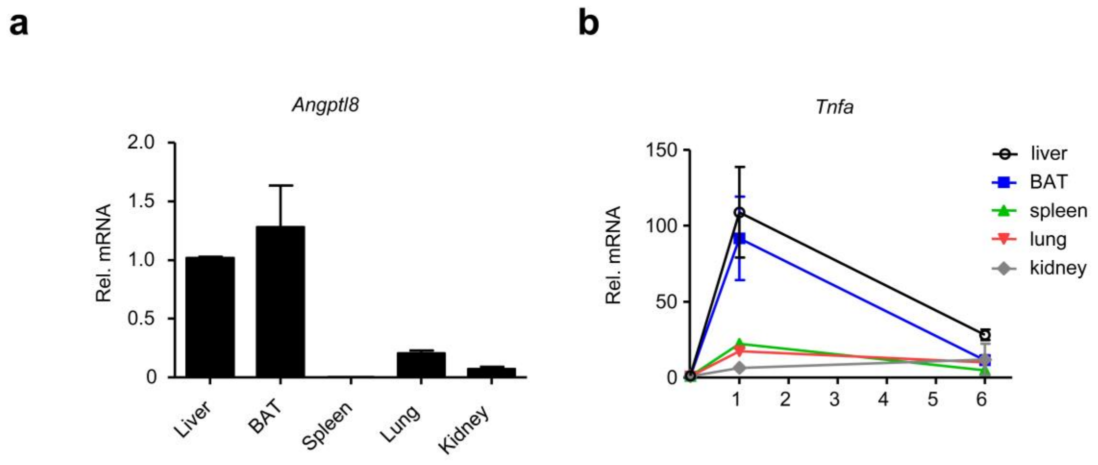

Supplementary Figure 11. Potential involvement of ANGPTL8 in acute inflammation caused by infection. **a.** The transcription profile of *Angptl8* in various tissues (n=3). **b.** The transcription of *Tnfa* in C57bl/6 mice after intraperitoneal injection of LPS (3 mg/kg). For 0 and 1 hours, n=3; for 6 hours, n=4. Data are shown as mean ± SEM, unpaired two-tailed student's test was used for statistics

Figure 1

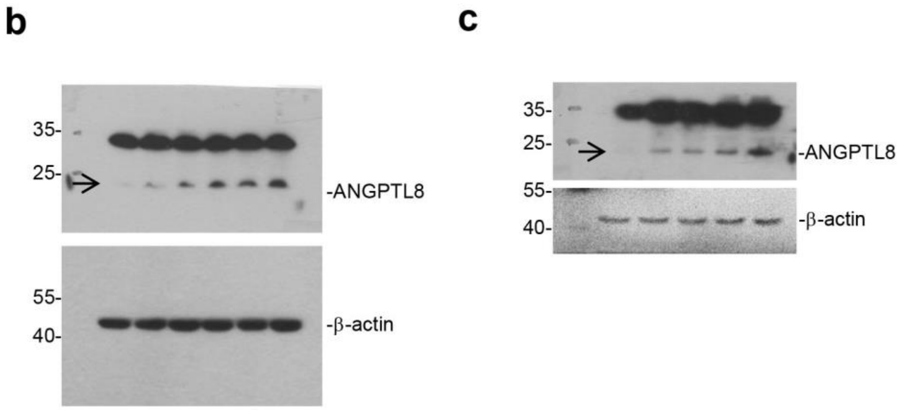

Figure 2

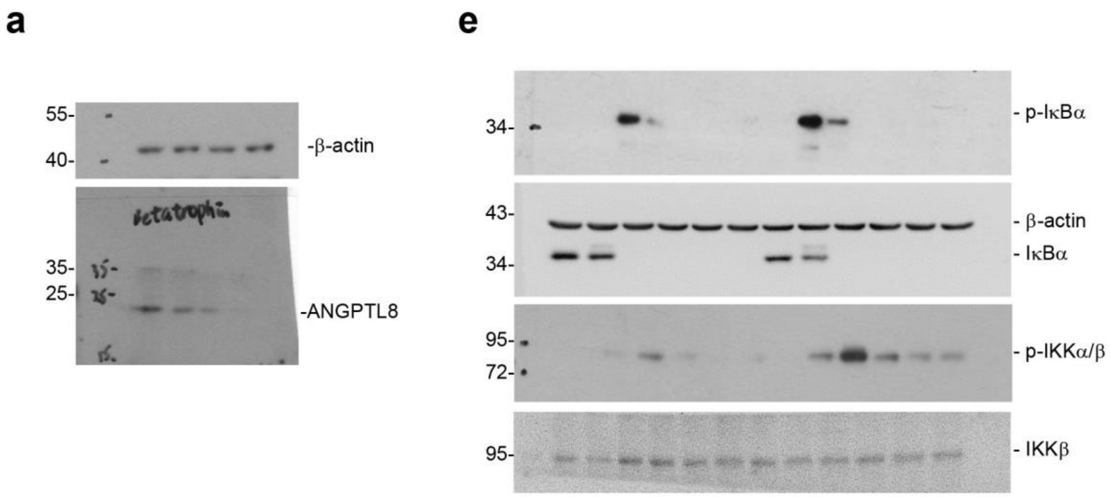

Figure 3

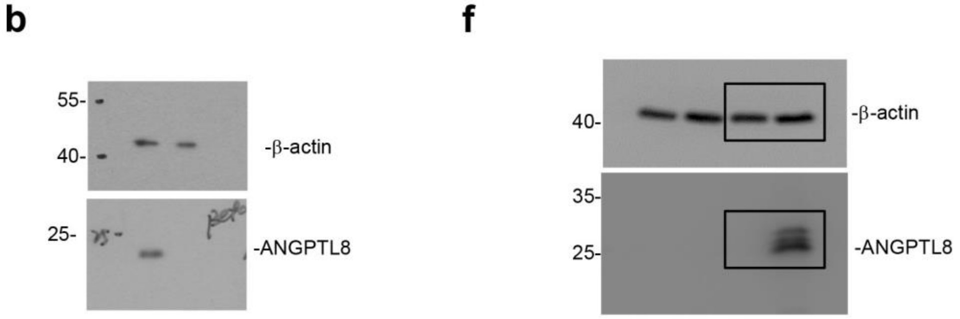

Supplementary Figure 12. The original immunoblots films for figure 1-3.

Figure 4

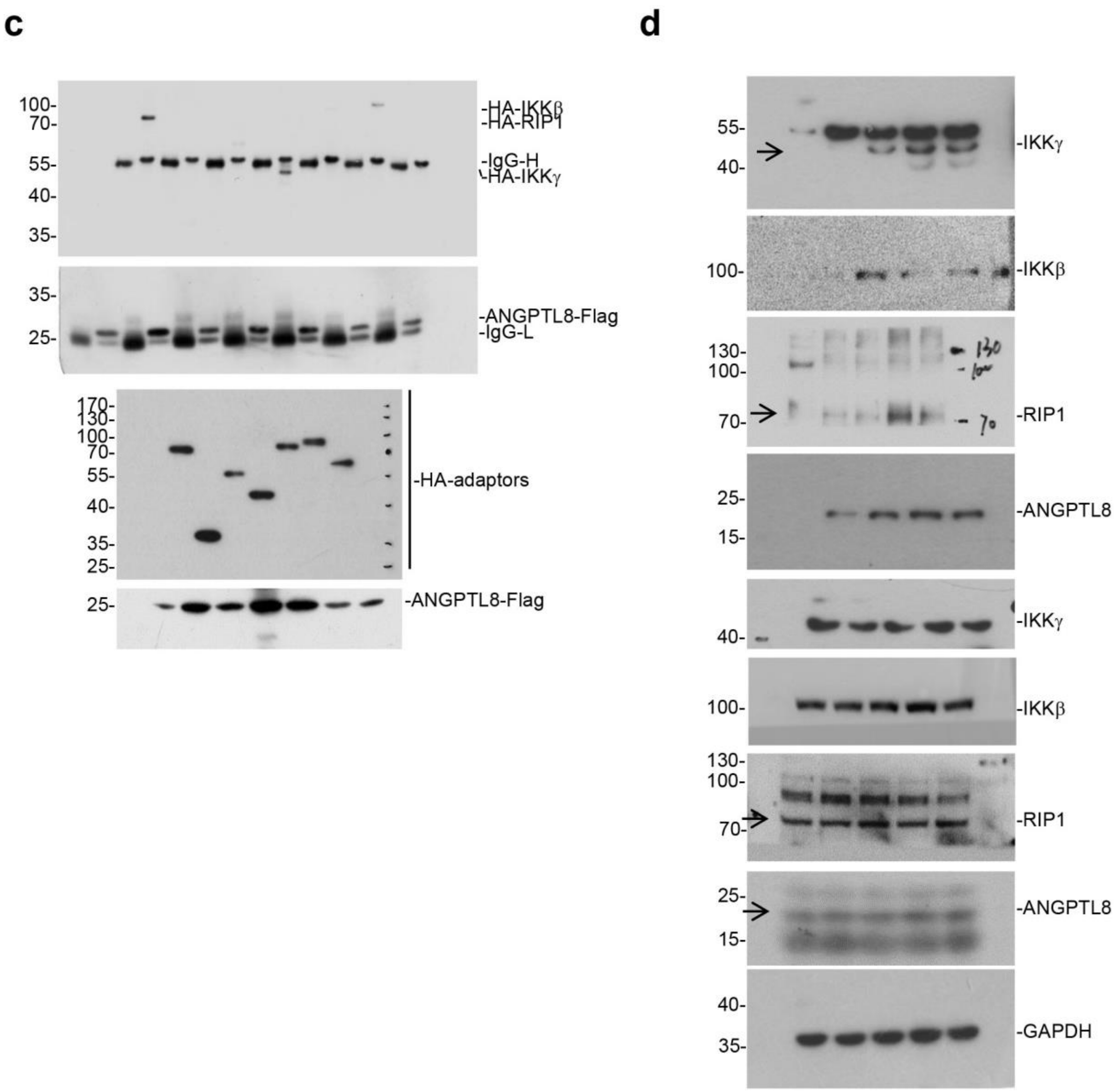

Supplementary Figure 13. The original immunoblots films for figure 4.

Figure 5

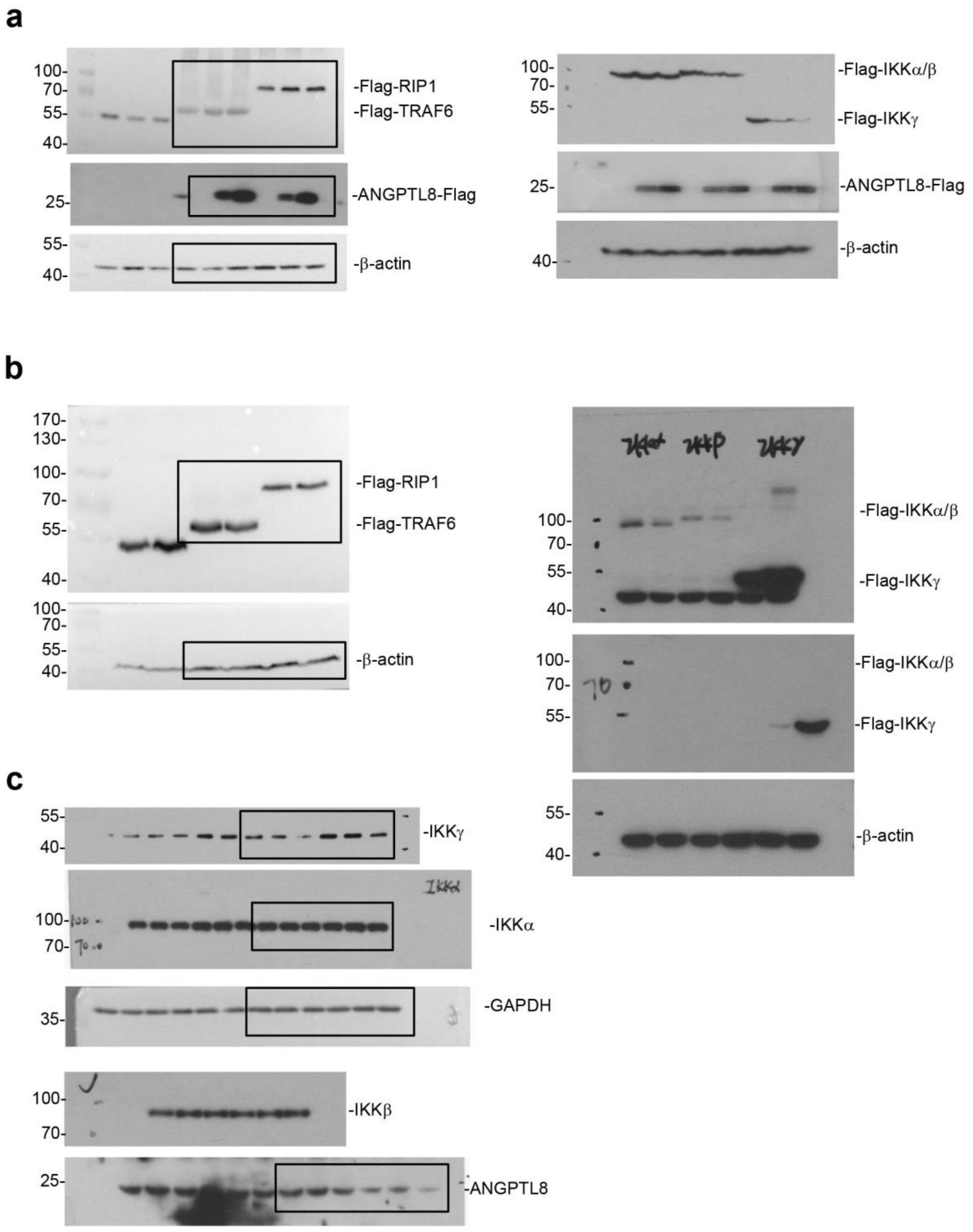

Supplementary Figure 14. The original immunoblots films for figure 5.

Figure 6

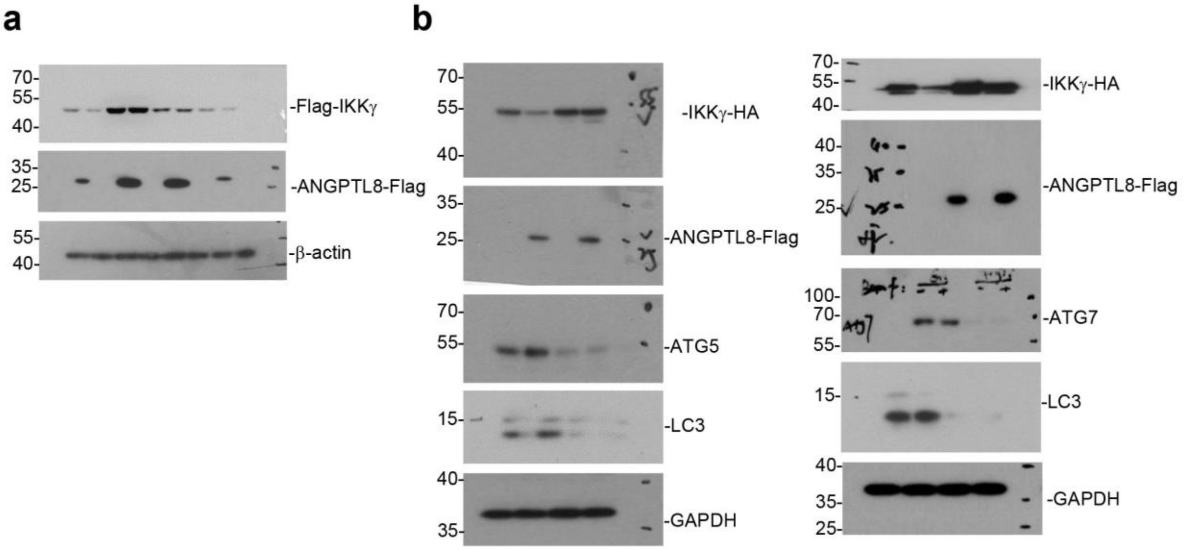

Figure 7

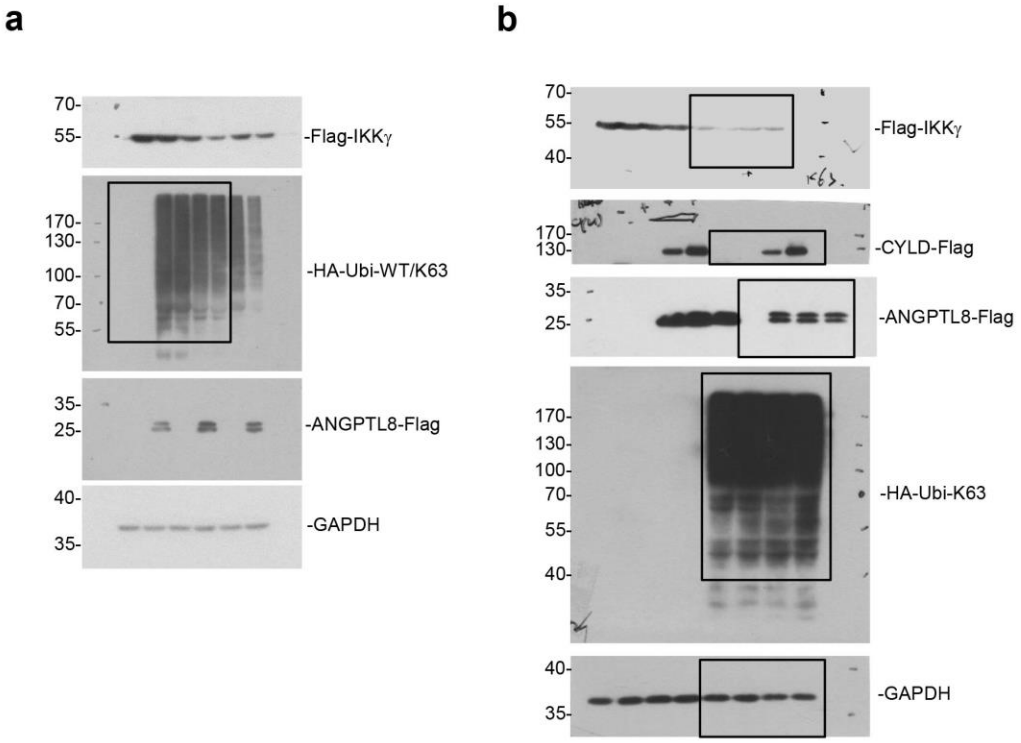

Supplementary Figure 15. The original immunoblots films for figure 6 and 7a,b.

Figure 7 (Continued)

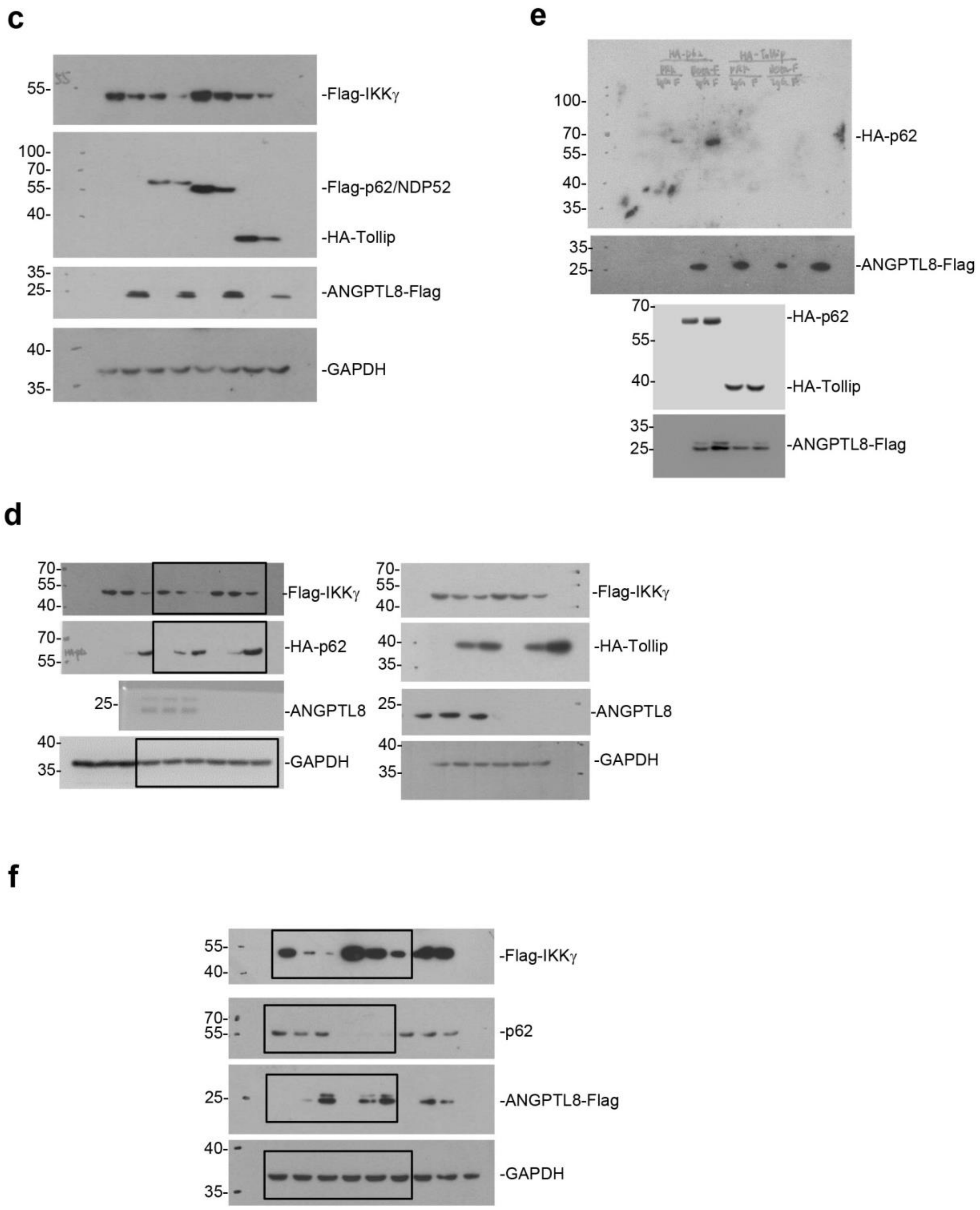

Supplementary Figure 16. The original immunoblots films for figure 7c,d,e,f.

Figure 8

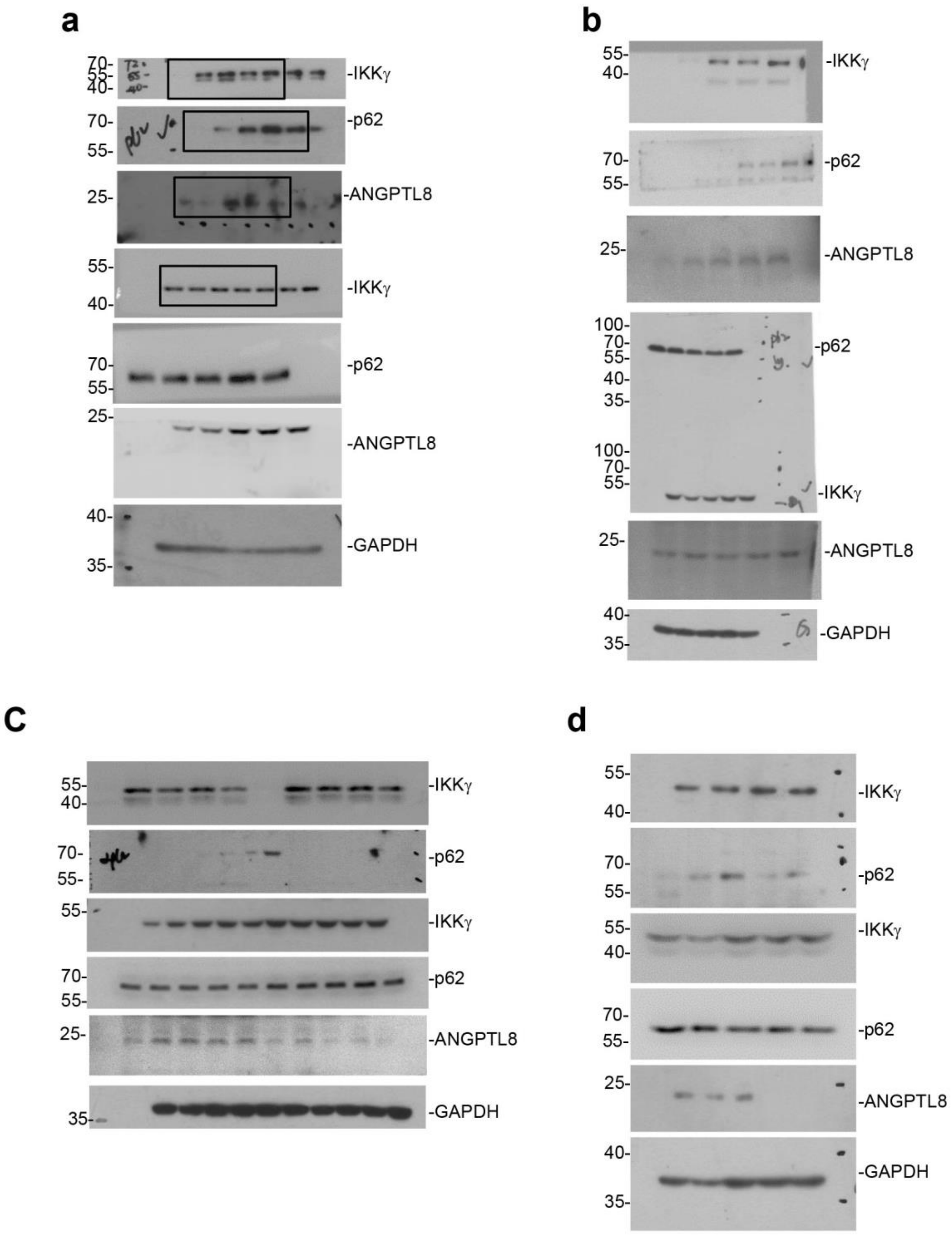

Supplementary Figure 17. The original immunoblots films for figure 8.

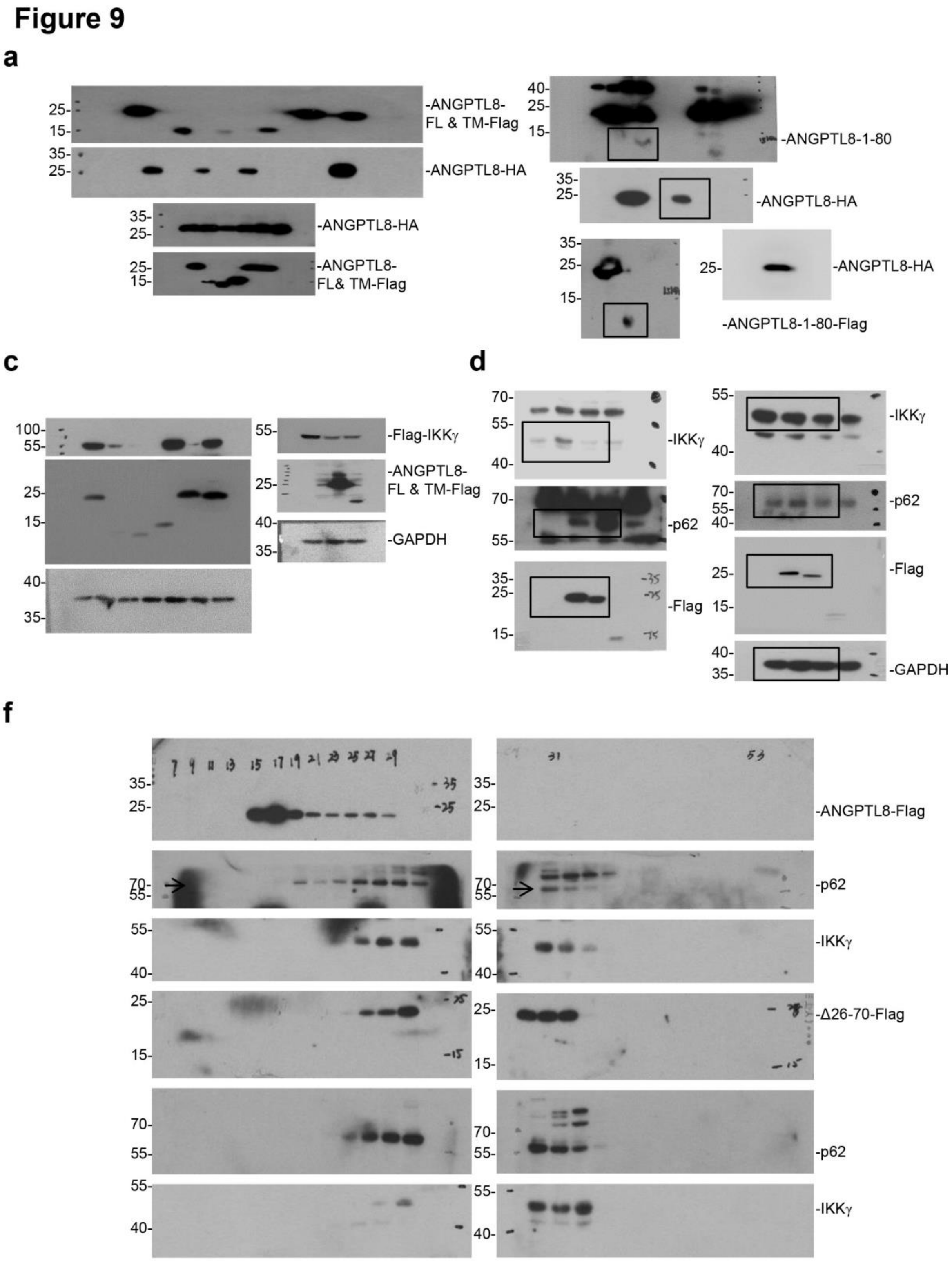

Supplementary Figure 18. The original immunoblots films for figure 9.

Figure 10

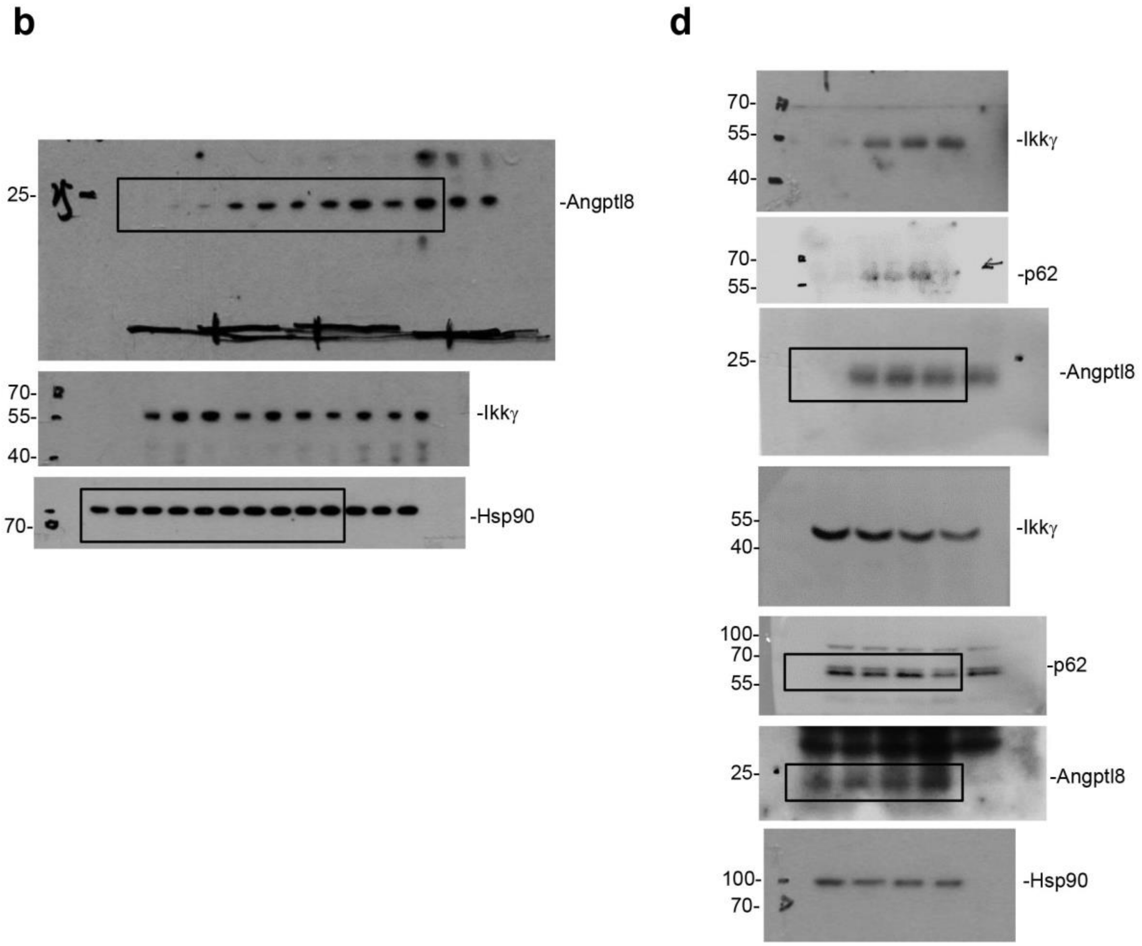

Supplementary Figure 19. The original immunoblots films for figure 10.

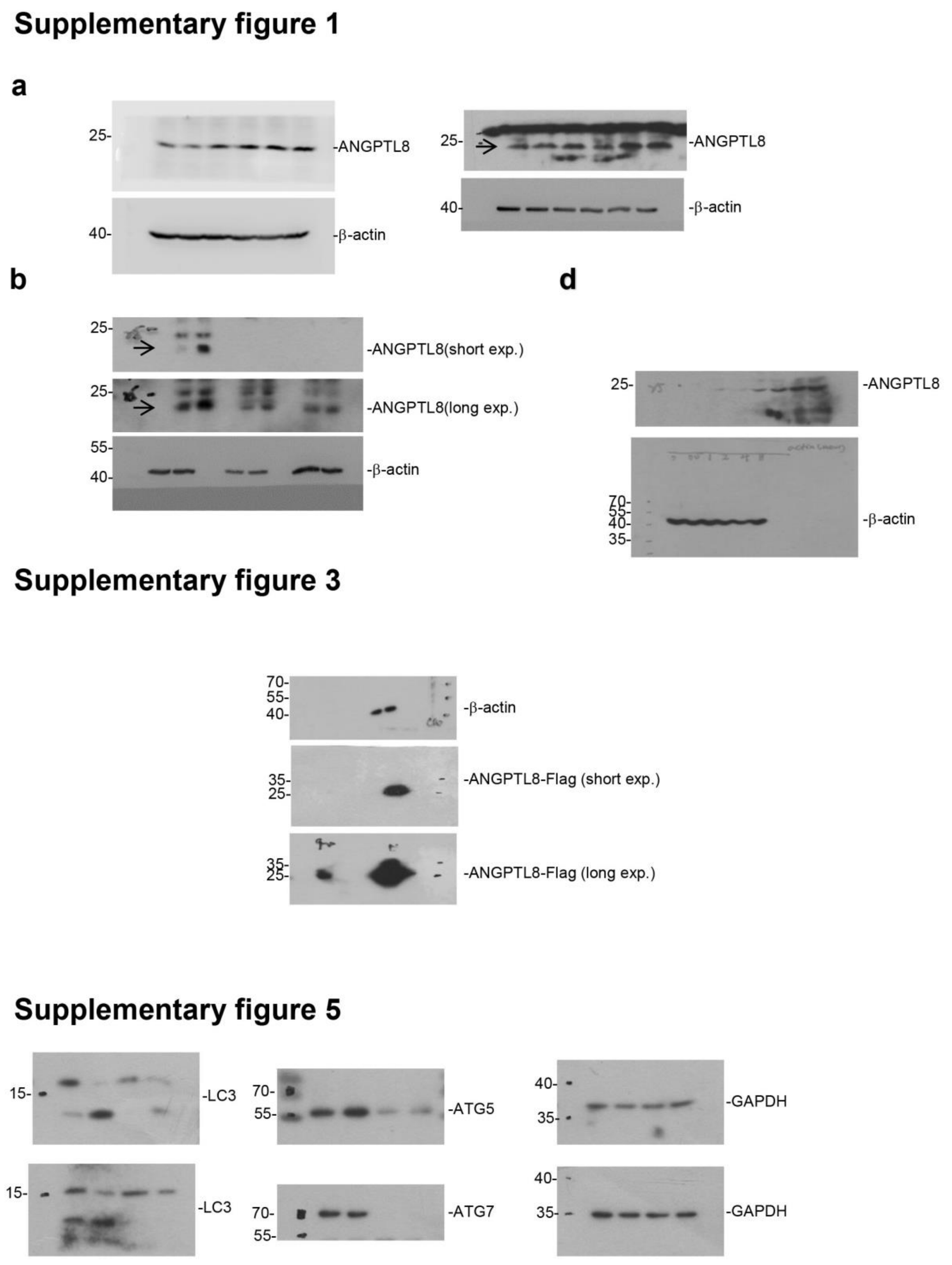

Supplementary Figure 20. The original immunoblots films for Supplementary figure 1,3,5.

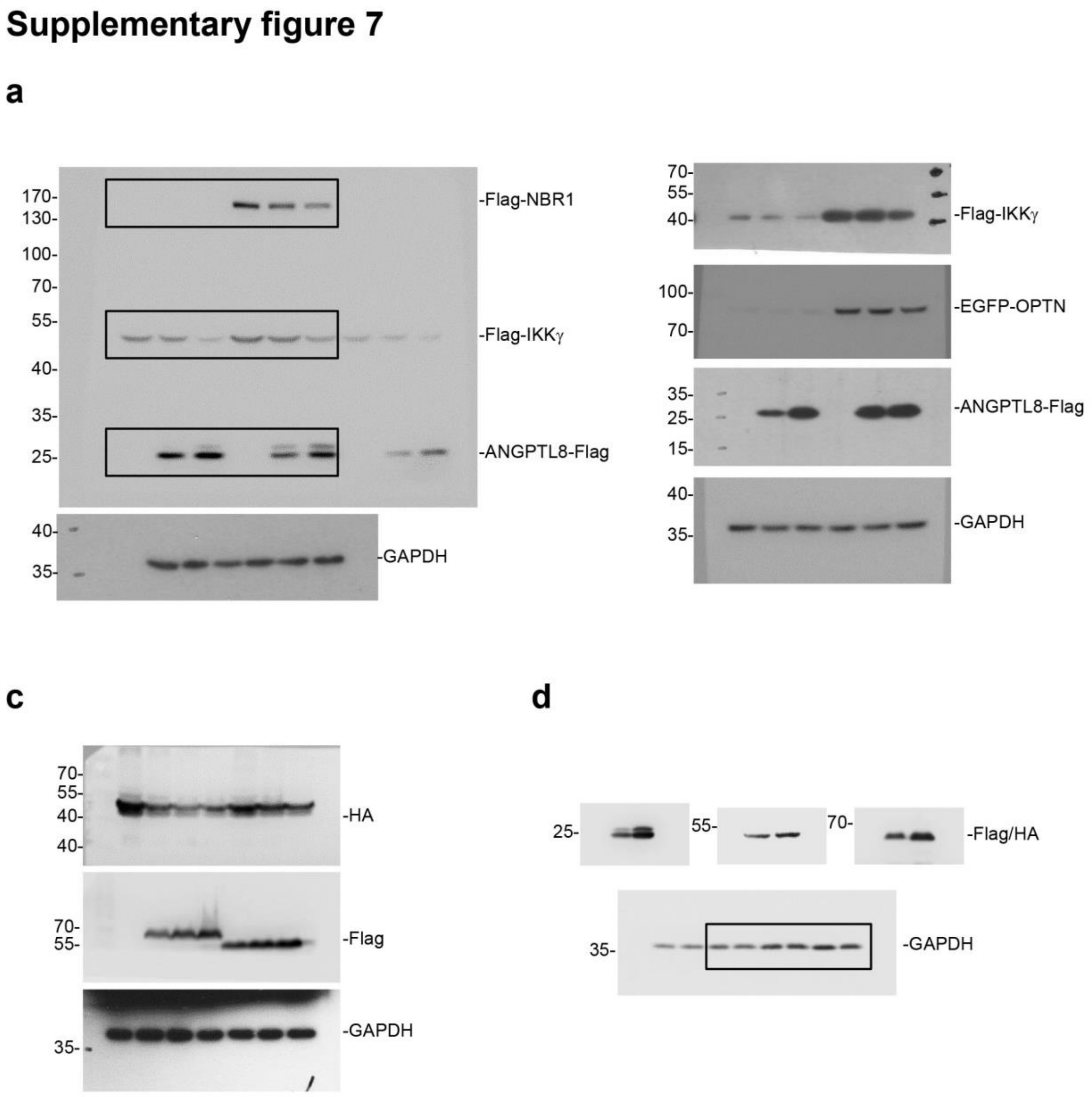

Supplementary Figure 21. The original immunoblots films for Supplementary figure 7.

Supplementary figure 8

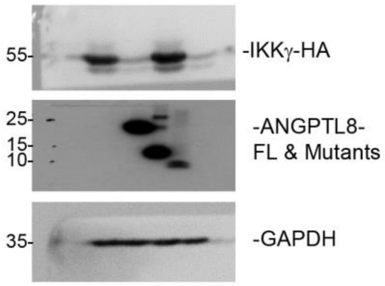

Supplementary figure 9

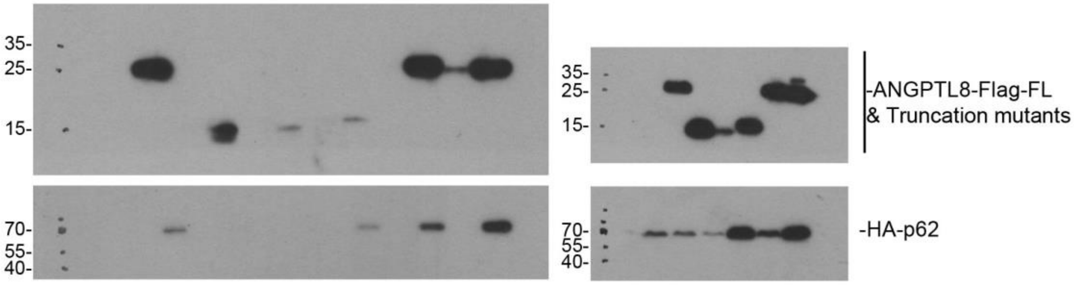

Supplementary figure 10

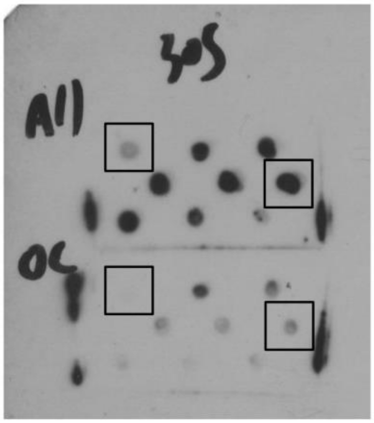

Supplementary Figure 22. The original immunoblots films for Supplementary figures 8-10.

Supplementary Table 1. Information of human blood samples

Healthy Control				Patients with acute inflammation											
**Serial NO.**	G	A	ANGPTL8 (pg/mL)	**Serial NO.**	G	A	Clinical diagnosis	PCT (mg/L)	ANGPTL8 (pg/mL)	**Serial NO.**	G	A	Clinical diagnosis	Endotoxin (EU/mL)	ANGPTL8 (pg/mL)
**N-01**	F	59	2773.87	**PCT-01**	M	52	Bacteremia	22.2	4137.63	**E-01**	M	73	Pneumonia	41.44	6382.23
**N-02**	M	46	902.50	**PCT-02**	M	53	Pneumonia	4.45	4325	**E-02**	M	93	Gastric ulcer with hemorrhage	0.85	16936.9
**N-03**	F	23	757.50	**PCT-03**	M	71	Herpes zoster	7.11	3187.5	**E-03**	M	73	Pneumonia	>50	11325
**N-04**	F	54	2912.95	**PCT-04**	M	52	Renal failure	16.2	6672.5	**E-04**	M	42	Acute upper gastrointestinal hemorrhage	1.14	8855
**N-05**	M	39	1552.50	**PCT-05**	F	61	Infective fever	0.93	8340.93	**E-05**	M	70	Severe pneumonia	3.14	11100
**N-06**	F	31	526.25	**PCT-06**	M	90	Cerebral insufficiency	0.82	5886.25	**E-06**	F	65	obstructive pulmonary emphysema	0.13	7305
**N-07**	F	45	1962.57	**PCT-07**	F	54	Pneumonia	8.48	8887.5	**E-07**	F	54	Pneumonia	27.37	15362.5
**N-08**	F	27	416.25	**PCT-08**	F	81	Pneumonia	0.57	8913.75	**E-08**	M	91	Pneumonia	0.22	8422.5
**N-09**	F	35	762.50	**PCT-09**	M	23	Pneumonia	0.64	7850	**E-09**	F	53	Acute renal failure	0.27	7950
**N-10**	F	43	1301.25	**PCT-10**	F	52	Brachial plexus neuritis	0.96	1738.75	**E-10**	M	91	Acute obstructive cholangitis	0.27	10807.5
**N-11**	F	60	1391.25	**PCT-11**	F	53	Renal failure	0.62	4850						
**N-12**	F	35	692.50	**PCT-12**	M	94	Acute obstructive cholangitis	11.83	6013.75						

Serial NO.	G	A	ANGPTL 8 (pg/mL)	Serial NO.	G	A	Clinical diagnosis	PCT (mg/L)	ANGPTL8 (pg/mL)	Serial NO.	G	A	Clinical diagnosis	Endotoxin (EU/mL)	ANGPTL8 (pg/mL)
**N-13**	M	22	947.50	**PCT-13**	F	61	Pneumonia; Renal failure	4.51	7067.5						
**N-14**	F	25	1305.00	**PCT-14**	F	79	Catagma	1.89	5443.75						
**N-15**	M	31	1021.25	**PCT-15**	M	58	Biliary tract infection	1.67	3112.5						
**N-16**	F	32	1213.75	**PCT-16**	M	70	Acute Gastric Mucosal Lesions	7.03	2646.25						
**N-17**	M	43	2932.27	**PCT-17**	F	76	Pneumonia; Chronic renal failure	27.51	6710						
**N-18**	F	35	801.25	**PCT-18**	M	91	Acute obstructive cholangitis	0.93	8422.5						
**N-19**	F	23	833.75												
**N-20**	F	33	813.75												
**N-21**	F	24	1153.75												
**N-22**	F	20	958.75												
**N-23**	F	31	1055.00												
**N-24**	M	43	1865.00												
**N-25**	F	53	1282.50												
**N-26**	M	36	683.75												
**N-27**	F	47	1688.75												
**N-28**	F	35	913.75												
**N-29**	F	51	1423.75												
**N-30**	M	45	1170.00												

Note: Blood samples were freshly collected and kept in -80℃ freezer till use. N: randomly chosen healthy subjects, whose blood were collected in the physical examination center; PCT: patients with positive detection of procalcitonin (>0.5mg/L); E: patients with positive detection of endotoxin (>0.1 EU/mL), a kind of lipopolysaccharide (LPS). NO. Number; G: gender; A: age

Supplementary Table 2. Reagents and antibodies used in this study

Antibodies/Reagents	Vendor	Catalog number	Dilution
Recombinant human TNFα	Novoprotein	C008	N/A
Recombinant humanIL-1β	PeproTech	200-01B	N/A
Recombinant human IFNγ	PeproTech	300-02	N/A
3-methyladenine, 3MA	Sigma	M9281	N/A
Chloroquine, CQ	Sigma	C6628-25g	N/A
MG132	Selleck	S2619	N/A
Flag	Sigma	F1804	1:10000
HA	Sigma	H3663	1:10000
p62	Sigma	655M4816V	1:5000
ANGPTL8	Sigma	SAB3501080	1:100
β-actin	Abcam	133626	1:10000
IKKγ	Abcam	ab178872	1:10000
GAPDH	Cell Signaling Technology	2118	1:10000
IκBα	Cell Signaling Technology	L35A5	1:5000
p-IκBα	Cell Signaling Technology	S32365A5	1:1000
IKKβ	Cell Signaling Technology	8943S	1:1000
p-IKKα/β	Cell Signaling Technology	C84E11	1:1000
IKKα	Cell Signaling Technology	2682S	1:1000
ATG5	Cell Signaling Technology	8540P	1:1000
ATG7	Cell Signaling Technology	2631P	1:1000
RIP1	Santa Cruz Biotechnology	SC7881	1:1000
Hsp90	BD	03717	1:5000
Oligomer-specific antibody	Merck Millipore	A-11	1:2000
Fibril-specific antibody	Merck Millipore	OC	1:2000

N/A, not applicable

Supplementary Table 3. Primers or target sequences for specific genes used in this study

**Gene Name**	**Primers/target sequences**
ANGPTL8-F	CCAGGCACAGAAGGTGCTAC
ANGPTL8-R	TGTGAGGGCCCATAGGATGT
IKKG-F	AGGTGGAGCACCTGAAGAGA
IKKG-R	CCTGGCATTCCTTAGTGGCA
Angptl8-F	CCCTCAATGGCGTGTACAGA
Angptl8-R	CCACCTGAATCTCCGACAGG
Tnfa-F	GGTGATCGGTCCCCAAAGGGATGA
Tnfa-R	TGGTTTGCTACGACGTGGGCT
ANGPTL8-gRNA	TGGTCCTGTACACACCGTTG
ANGPTL8-identification-F	CGGCCAGTTAACGATTGAC
ANGPTL8-identification-R	GTGGTCCTGTACACACCGTTG
ANGPTL8-RNAi-#1	CTGACAAAGGCCAGGAACAT
ANGPTL8-RNAi-#2	CTCAGATGGAGGAGGATAT
ANGPTL8-RNAi-#3	GACAGATCCAGGAGAGACT
p62-RNAi	CTGGACCCATCTGTCTTCA
IKKγ-RNAi	GAATGCAGCTGGAAGATCT
ATG5-RNAi	GAAGCAGAACCATACTATT
ATG7-RNAi	GGAGTCACAGCTCTTCCTT
